# The potential role of rutin, a flavonoid, in the management of cancer through modulation of cell signaling pathways

**DOI:** 10.1515/biol-2025-1181

**Published:** 2025-10-18

**Authors:** Hajed Obaid A. Alharbi, Shehwaz Anwar, Arshad Husain Rahmani

**Affiliations:** Department of Medical Laboratories, College of Applied Medical Sciences, Qassim University, Buraydah, 51452, Saudi Arabia; Department of Medical Laboratory Technology, Mohan Institute of Nursing and Paramedical Sciences, Bareilly, 243302, India

**Keywords:** rutin, oxidative stress, inflammation, cancer therapy, pathogenesis, bioavailability

## Abstract

Cancer treatment continues to face challenges due to adverse effects, drug resistance linked with conventional therapies, and high costs. As increasing interest in safer and cost-effective alternatives drugs, natural products such as flavonoids have been explored for treating cancer. Rutin, a dietary flavonoid, exhibits diverse pharmacological activities that may contribute to cancer prevention and treatment. It interferes with cancer progression by inducing apoptosis and autophagy, promoting cell cycle arrest, regulating oxidative stress, activating tumor suppressor gene, and modulating various signaling cascades. Recent studies also suggest that combining rutin with other therapeutic agents or employing nanoformulations may enhance its bioavailability and anticancer efficacy. This review critically examines anticancer mechanisms across various cancer types and highlights novel strategies to explored their therapeutic potential. The comprehensive clinical trials and mechanistic studies are needed to validate its safety, bioavailability, and efficacy in cancer management.

## 1 Introduction

With rising incidence and fatality rates in recent years, cancer has become a primary global health concern. Despite advancements in many therapeutic modalities, cancer remains a leading cause of mortality globally [1,2]. Although current cancer therapies offer excellent benefits, these medicines are associated with severe side effects, including extreme nausea and vomiting [3]. Thus, there is a need for more effective, cost-effective, and safer treatment approaches to manage cancer. Numerous herbs are being studied for their tumoricidal properties against various types of cancer. Many of these herbs have undergone clinical trials. New strategies are being developed that combine molecular targeting of cancer pathways with cytotoxic therapies [4]. Continuous research and development have been aimed at discovering new cytotoxic compounds from medicinal plants with anti-proliferative activity [5]. Numerous medicinal plants have been demonstrated to aid in the prevention and treatment of a variety of ailments. People have utilized plants as remedies to treat a wide range of diseases throughout history. This is because a number of bioactive compounds found in plants can be used to treat a wide range of diseases [6]. Numerous therapeutically important substances for the development of new drugs have been found in natural products and secondary metabolites [7]. They are also reasonably non-toxic, affordable, and available in an ingestible form [8]. These naturally occurring substances have been demonstrated to meaningfully contribute to the prevention as well as treatment of disease in various pathologies, especially cancer [9,10,11,12].

**Figure 1:**
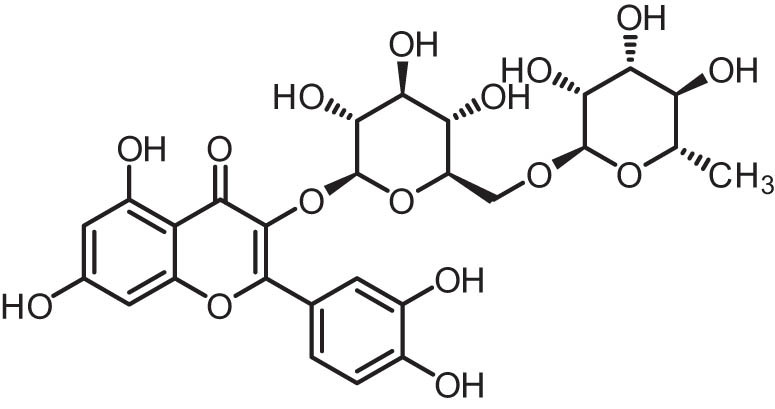
Chemical structure of rutin (drawn by ChemDraw Professional 15.0).

**Figure 2:**
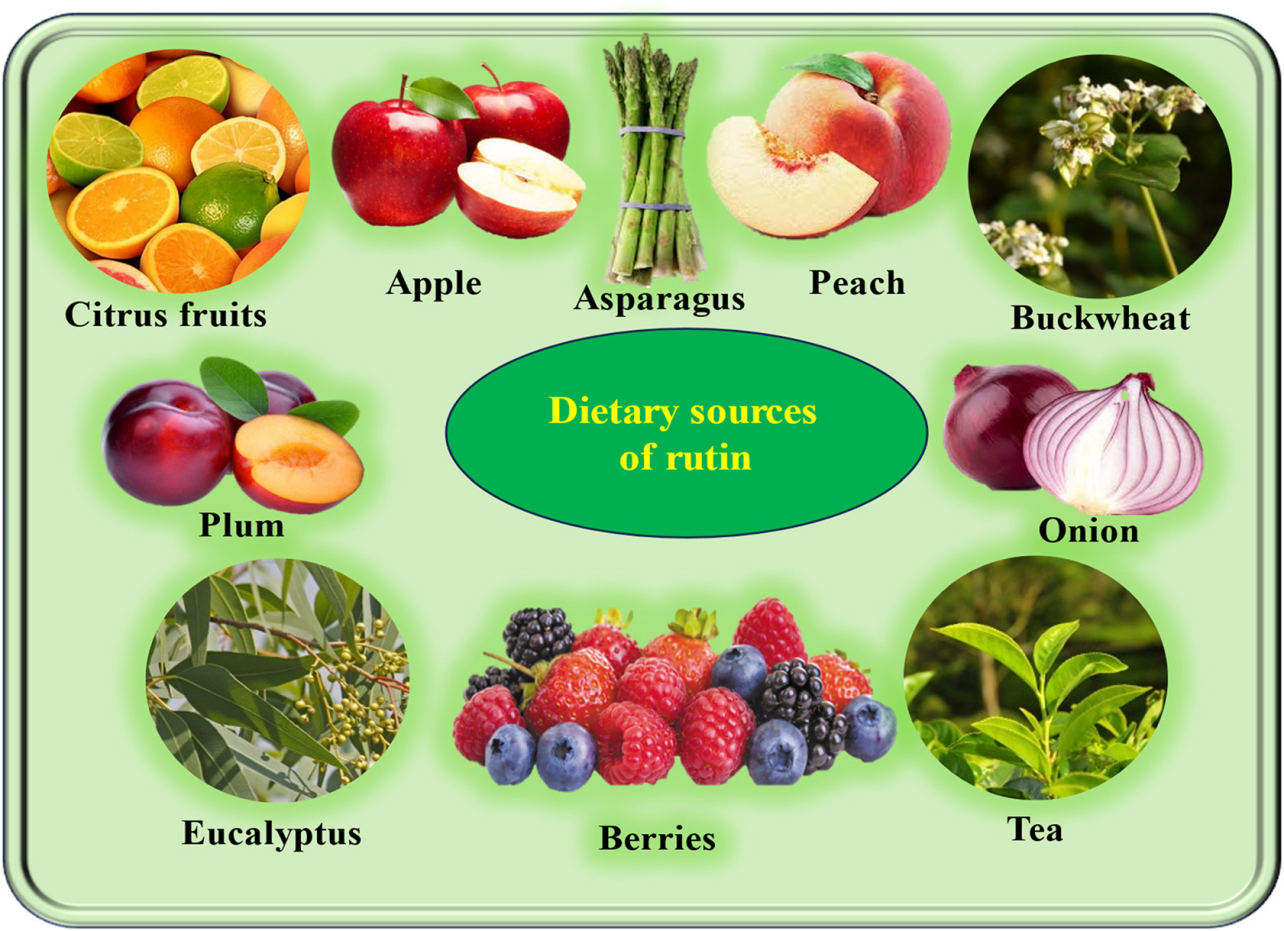
Dietary sources of rutin. Rutin is a plant-derived flavonoid abundantly found in a variety of natural food sources such as citrus fruits, apples, asparagus, peaches, buckwheat, onions, tea, plums, berries, etc.

Additionally, plants have a variety of flavonoids, which are excellent sources of antioxidants and can suppress the pathogenesis of numerous diseases. Moreover, several plant-based chemotherapy medicines have demonstrated appreciable enhancements in the anti-cancer activity of several chemotherapeutic agents [13,14,15]. Rutin (3′,4′,5,7-tetrahydroxy-flavone-3-rutinoside) (Figure 1) is found in fruits, vegetables, and medicinal plants (Figure 2) and is an important nutritional component of many foodstuffs [16,17]. Rutin is best known for its antioxidant and anti-inflammatory effects. These potential benefits of rutin are essential factors implicated in the prevention of numerous pathogeneses. Previously, rutin exerts several health-promoting effects by modulating key molecular pathways [18].

Numerous investigations have revealed that rutin modulates several cell signaling molecules, such as those involved in apoptosis, the cell cycle, angiogenesis, and autophagy, and so plays a significant role in managing cancer [19,20,21]. Therefore, this review explores rutin’s potential as an anticancer agent through a detailed analysis of these modulatory effects.

## 2 Search strategy

This review article discusses the role of rutin in various types of cancer. The review gathered a wide-ranging literature search based on English-language publications using PubMed, Google Scholar, and Scopus databases. They used keywords such as “rutin and cancer,” “rutin and inflammation,” “rutin and oxidative stress,” “rutin and angiogenesis,” “rutin and STAT-3,” “rutin and Akt/PI3K,” “rutin and breast cancer,” “rutin and lung cancer,” “rutin and cervix cancer,” “rutin and liver cancer,” “rutin and gastric cancer,” and “rutin and leukemia.” The review also explores the synergistic effects of rutin with other compounds.

## 3 Major mechanisms of rutin involved in cancer management

Natural products and their active compounds have been reported for their anti-inflammatory potential that helps prevent or mitigate the progression of different disease pathogeneses [22,23,24,25]. Rutin has demonstrated its anticancer properties across diverse types of cancer by modulating essential molecular and cellular pathways, as evidenced in both *in vivo* and *in vitro* studies (Table 1). The following are some of the main approaches through which rutin manages cancer.

**Table 1:** Mechanism of action of rutin in cancer

Action on	Cancer	Study types	Doses	Findings of the study	Refs.
Inflammation	Breast cancer	*In vivo*	100 mg/kg	Decreased iNOS and NF-kB, increased antioxidant levels, inhibited inflammatory cytokines	[30]
	Brain cancer	*In vitro*	30 µM	Modulated inflammatory mediators	[32]
Endoplasmic reticulum	Breast cancer	*In vitro*	40 µM	Induced apoptosis via ER stress with doxorubicin	[37]
P53	Brain cancer	*In vitro*	0–20 M	Apoptosis via upregulation of P53; Apoptosis was reversed by P53 knockdown	[41]
	Prostate cancer	*In vitro*	700 µM	Increased expression of the p53 gene	[42]
		*In vitro*	100 and 150 µM	Increased expression of p53 and Bax	[44]
Oxidative stress	Breast cancer	*In vitro*	100 mM	Reversed ROS increase	[57]
	Lung and colon cancer	*In vitro*	5–100 mM	Dose-dependent reduction of superoxide anion	[58]
	Breast cancer	*In vivo*	20 mg/kg	Increased GSH and decreased MDA level content	[59]
Apoptosis	Brain cancer	*In vitro*	0, 1, 5, and 10 µM	Induced apoptosis	[41]
		*In vitro*	25, 50, 100 µM	Downregulated BCL2/BAX ratios, Induced apoptosis	[65]
	Pancreatic cancer	*In vitro*	5 and 10 μg/mL	Induced apoptosis, and apoptosis-related protein expression	[66]
Cell cycle	Breast cancer	*In vitro*	20 μM and 50 μM	Increased the rate of cell death.	[71]
	Brain cancer	*In vitro*	50–100 μM	Inhibited cell viability and proliferation, reduced ERK1/2 phosphorylation, G2 arrest	[73]
	Brain cancer	*In vitro*	0, 25, 50, and 100 μM	Cells in the G2/M phase accumulation	[65]
Angiogenesis	Human promyelocytic leukemia	*In vitro*	10 and 30 μM	Decreased VEGF secretion	[80]
	Breast cancer	*In vitro*	25, 50, and 100 µM	Decreased Bcl2 and VEGF in a dose-dependent manner via vanadium-rutin treatment	[44]
	Colon cancer	*In vitro*	1, 10, 20 mg/kg	Decreased VEGF level	[83]
	Breast cancer	*In vitro*	200 µM	Induced angiogenesis by downregulating the anti-angiogenic marker	[85]
AP-1	Human promyelocytic leukemia	*In vitro*	10 and 30 µM	Rutin and VE together inhibited the binding actions of AP	[80]
Autophagy	Liver cancer	*In vitro*	75 μM	Reduced autophagy and BANCR expression	[95]
	Oral & lung cancer	*In vitro*	0–40 μM	Stimulated autophagy	[96]
	Brain cancer	*In vitro*	100 or 200 μM	TMZ increased the expression of LC3II, whereas rutin treatment reversed this effect	[97]
PI3K/AKT/mTOR	Ehrlich ascites carcinoma	*In vivo*	25 mg/kg	Reduced expressions of PI3K, AKT, mTOR	[100]

### 3.1 Anti-inflammatory effects

Chronic inflammation is an important driving force behind tumor initiation and progression [26,27,28]. Rutin has demonstrated potent anti-inflammatory properties through the modulation of several signaling pathways implicated in cancer development. In the lipopolysaccharide-induced mastitis mouse model, rutin was shown to alleviate inflammation by inhibiting the nuclear factor kappa B (NF-κB) signaling pathway and attenuating endoplasmic reticulum (ER) stress, revealing its ability to disrupt pro-inflammatory responses [29]. Another study using a 7,12-di-methylbenz(a)anthracene (DMBA)-induced breast cancer model of female rats, highlighted rutin’s impact on inflammatory signaling by downregulating interleukin 6/NF-κB, and modulating SRC1/HSP90, and ER-α signaling pathways, ultimately suppressing tumor biomarkers, including Carcinoma Antigen 15-3, proto-oncogene Tyrosine-Protein Kinase Src1 and Inducible Nitric Oxide Synthase (iNOS) [30]. Importantly, this work links anti-inflammatory modulation directly with tumor suppression.

In mice with the human papillomavirus type 16 (HPV16) transgene, rutin reduced cyclooxygenase-2 (COX2) expression in both epidermis and dermis. Co-treatment with rutin and curcumin decreased tumor-associated inflammation, suggesting that these nutritional substances could regulate COX2 expression in HPV16-induced lesions by limiting leukocytic infiltration [31]. In glioblastoma (GBM) microenvironment, rutin indirectly modulated inflammatory signaling by altering microglial miRNA-125b and STAT3 expression in co-culture with cells of GBM. Rutin significantly reduced the viability of GL15 cells [32]. The K14-HPV16 mouse model, commonly used to study the wasting syndrome associated with HPV-induced cancers, demonstrated that rutin exerted strong muscle-protective effects through NF-κB downregulation [33].

In summary, the anti-inflammatory potential of rutin is a major mechanism in the suppression of cancer, particularly through targeting NF-κB, signal transducer and activator of transcription 3 (STAT-3), and COX-2 pathways in tumor-related inflammatory environments.

### 3.2 Modulation of ER stress

Tumor cells often encounter microenvironmental stress, leading to the disruption of the functions of the ER. ER stress response has been reported to disturb intracellular homeostasis and impair normal cell function [34]. ER stress may help tumor cells return to homeostasis and foster an environment conducive to tumor survival and growth [35]. Cancer cells are known for their capacity to spread locally or metastasize into neighboring tissues. Unfavorable circumstances in these novel settings, such as low glucose, hypoxia, growth factor deprivation, oxidative stress, lactic acidosis, and amino acid starvation, can impair the endoplasmic reticulum’s ability to fold proteins correctly [36]. One finding focused on the role of ER stress in apoptotic cell death. The disruption of ER activities plays a critical role in triggering apoptosis. The study shows that combinational treatment with rutin and doxorubicin significantly enhances ER stress-mediated apoptotic cell death in triple-negative breast cancer cells [37]. Recently, the effects of rutin at different concentrations (400–700 mM/mL) on the cell cycle, metabolism, and apoptosis of human colon cancer cells (SW480) were investigated. The enrichment analysis of the miRNAs–lncRNAs–mRNAs–TFs network in these cells suggested that rutin may modulate ER stress [38]. Collectively, modulation of ER stress by rutin contributes significantly to apoptosis and growth inhibition in cancer cells, making it a valuable component of combinational approaches for cancer management.

### 3.3 Activation of p53 gene

The gene p53 protects the genome and suppresses tumors. It is essential in deciding how cells react to stimuli that cause DNA damage [39]. This well-known tumor suppressor gene induces apoptosis and arrest of the cell cycle [40]. Through boosting p53 activity, several natural compounds have been shown to combat cancer. In glioma CHME cells, rutin induced the marked cytotoxicity and cell death by p53-mediated apoptosis. It upregulated BAX, promoted cytochrome c release, activated caspases 3 and 9, and downregulated BCL. Moreover, apoptosis induced by rutin was reversed in a concentration-specific way when p53 was knocked down, confirming a p53-dependent mechanism [41].

The effects of rutin and 5-fluorouracil (5-FU) combination were investigated on prostate cancer cells. The combined administration of 5-FU and rutin increased both apoptosis and expression of p53 genes in PC3 cells, a cell line used to model prostate cancer. Moreover, it was concluded that the synergistic potential of the 5-FU/rutin combination on this cancer cell line improved the expression of the p53 gene, downregulation of Bcl-2 protein, and apoptosis compared to control separate applications [42]. In contrast to tamoxifen, in MCF-7 cells, genes associated with the cell cycle (CDK1 and p21) and both tumor suppressors (p53 and PTEN) were upregulated by rutin; using a p53-dependent mechanism, this compound caused G2/M arrest and apoptosis of the cells of MCF-7 [43].

A study expertly focused on many apoptotic pathways in human breast cancer cell lines to investigate the vanadium–rutin complex’s involvement in curing cancer. The complex’s chemotherapeutic properties were evidenced by activating p-53-dependent intrinsic apoptosis and regulating the vascular endothelial growth factor (VEGF) cascades. The complex effectively began apoptosis by activating the p53-dependent intrinsic apoptotic route in both kinds of cells [44]. Thus, the ability of rutin to modulate p53-dependent intrinsic apoptosis pathways underscores its potential as an effective anticancer compound targeting genomic integrity regulators.

### 3.4 Antioxidant potential

The accumulation of reactive oxygen species (ROS), including superoxide ions, is a sign of oxidative stress. ROS are signaling molecules that regulate vascular smooth muscle cell growth, contraction, and relaxation [45]. Oxidative stress is a critical factor implicated in several pathologies [46], including diabetes mellitus and neurodegenerative diseases, and it plays a dual role in cancer, contributing to genetic mutations and tumorigenesis at moderate levels. Cancer cells show abnormal redox homeostasis, whereas ROS are pro-tumorigenic, and higher levels of ROS are cytotoxic [47,48]. The management of ROS concentrations has been considered an auspicious therapeutic approach, mainly focusing on the anticancer role of natural products [49]. Additionally, numerous epidemiological studies showed a negative correlation between a diet rich in fruit/vegetables and cancer occurrence or progression [50,51]. Flavonoids protect plants against microbial infections, ultraviolet radiation, and oxidative stress [52].

Dietary nutrients can enhance the body’s natural antioxidant defenses by scavenging ROS [53]. Rutin has been demonstrated to act as a neuroprotectant in ischemic animal models and reperfusion-induced cerebral injury [54,55,56]. In one study, olmesartan exhibited cytotoxic activity against HeLa and MCF-7 cell lines when administered following rutin pretreatment. The observed decrease in ROS levels suggested decreased viability of cells treated with olmesartan [57]. Another study examined the effects of rutin on human lung (A549) and colon (HT29 and Caco-2) cancer cell lines, focusing on migration, viability, adherence, and superoxide anion formation. As per the outcomes, rutin treatment prevented the viability of malignant cells and reduced the formation of superoxide in HT29 cells. Furthermore, rutin impaired the migration and adherence of HT29 and A549 cells [58]. In a separate study, both rutin and orlistat showed anticancer efficacy in two breast carcinoma models (*in vivo* EAC and *in vitro* MCF7) as well as in pancreatic cancer (PANC-1) cell lines. Lowering of tumor volume, fatty acid synthase expression, cholesterol, carcinoembryonic antigen (CEA) level, along with increased antioxidant capacity (elevated GSH and lowered malondialdehyde (MDA) content), and histological improvements supported the anticancer effect [59]. Therefore, by restoring redox balance and inhibiting oxidative stress, rutin exhibits strong protective effects against cancer initiation and progression.

### 3.5 Induction of apoptosis

Apoptosis is a tightly regulated cellular process characterized by condensation of chromatin, shrinkage of cells, fragmentation of chromosomal DNA, blebbing of the cell membrane, and fragmentation of the nucleus [60]. Various diseases, including cancer, can arise from the disruption of the balance among homeostasis and cell growth and apoptosis. Unchecked apoptosis is a key mechanism underlying tumorigenesis [61]. There are two mechanisms, including extrinsic and intrinsic signaling mechanisms, for apoptosis [62]. Despite diverse origins, all types of cancer share common characteristics, such as unchecked growth, angiogenesis, and evasion of apoptosis [63,64]. Therefore, therapies based on natural compounds have shown promise in modulating these cancer hallmarks. In 2018, rutin and orlistat exhibited cytotoxicity against MCF-7 and Panc-1 cell lines by inducing apoptosis [59]. Rutin was also shown to induce cytotoxicity in CHME cells and cause cell death by inducing apoptosis through the upregulation of p53. Rutin caused nuclear condensation, membrane blebbing, and fragmentation, and increased oxidative stress. It further promoted apoptosis by stimulating the release of cytochrome c, downregulating BCL, upregulating BAX, and activating both caspase 9 and caspase 3 [41]. Through inducing cell death and G2/M arrest in the cell cycle progression, as well as controlling the expression of genes linked to apoptosis, it also showed notable anti-neuroblastoma actions in human neuroblastoma cell lines. These findings suggested that rutin has significant anti-neuroblastoma properties by boosting cell death, triggering G2/M arrest in cell cycle progression, and modulating the expression of genes linked to apoptosis [65]. Additionally, rutin promoted apoptosis in pancreatic cancer cells by elevating the production of miR-877-3p, which in turn suppressed Bcl-2 transcription. It is concluded that miR-877-3p mimics and rutin may increase the production of apoptotic proteins [66]. Rutin-induced apoptosis has been linked to caspase-3 activation and a lowering in mitochondrial membrane potential (MMP) [67]. Overall, rutin effectively induces apoptosis via mitochondrial dysfunction and caspase activation, positioning it as a potent natural apoptotic inducer in cancer therapy.

### 3.6 Cell cycle arrest

Disruption of the cell cycle checkpoints is central to tumor progression, as cell cycle alterations contribute to uncontrolled cell proliferation and cancer growth [68,69]. Additionally, inducing cell cycle arrest at various checkpoints has been reported to be a promising anticancer strategy [70]. Rutin exhibits its anticancer potential through cell cycle arrest, and studies prove it. In MCF-7 cells, it upregulated the expression of cell cycle-related regulators like p21 and CDK1, as well as key tumor suppressors, including p53 and PTEN, in contrast to tamoxifen. This compound induced G2/M arrest and apoptosis in MCF-7 cells through a p53-dependent pathway [43].

Interestingly, rutin has been reported to increase the sensitivity of cancer cells to cyclophosphamide and methotrexate, while also reversing multidrug resistance. It also induces cell cycle arrest at both G2/M and G0/G1 phases, thereby promoting apoptotic cell death [71]. In another study focused on lung cancer, the combination of rutin and cisplatin significantly increased the number of apoptotic cells as compared to the untreated control group. The results suggest that rutin promotes the tumor necrosis factor-α (TNF-α)-induced apoptosis of A549 human lung carcinoma cells [72]. Rutin has been found to have pro-apoptotic and antiproliferative effects on human GBM cell lines (GL-15). In addition, it arrests the cell cycle at the G2 stage and promotes the differentiation of GL-15 cells into an astroglial phenotype [73]. In 2013, research was published on the anti-tumor effect of rutin on human neuroblastoma cell lines. Rutin reduced LAN-5 cell proliferation and chemotactic potential. It caused cell death and G2/M arrest in the cell cycle progression, as shown by flow cytometry analysis [65]. Rutin was reported to show anticancer activity in HPV-C33A cervical cancer cells through G0/G1 cell cycle arrest and induction of apoptosis [67]. Therefore, rutin’s ability to modulate cell cycle progression contributes significantly to its anti-proliferative and cytostatic effects against diverse cancers.

### 3.7 Inhibition of angiogenesis

The over-expression of pro-angiogenic factors and the suppression of anti-angiogenic factors lead to aberrant and excessive angiogenesis in various non-neoplastic angiogenic diseases [74,75,76]. The “angiogenic switch” process in tumors marks the onset of the creation of blood vessels, which is triggered when pro-angiogenic signaling dominates over anti-angiogenic control [77]. Additionally, angiogenesis aids in advancing several malignant cancers [78,79].

It has been demonstrated that natural components can help inhibit cancer progression through limiting angiogenesis. According to Chuang et al., a combination of vitamin E and rutin suppresses VEGF expression in HL-60 cells by the downregulation of insulin-like growth factor 1 receptor (IGF1-R)/insulin receptor substrate-1 (IRS-1) protein production, mediated by reduced activator protein 1 (AP-1) interaction activity [80]. Similarly, the flavonoids identified in *Murraya koenigii* leaf extract, including quercetin, rutin, kaempferol, and apigenin, reduced the activity of the endogenous 26S proteasome in MDA-MB-231 cells in a dose-dependent way. The lower expression of angiogenic and anti-apoptotic gene markers in the leaf extract-treated tumors indicates that angiogenesis is inhibited and apoptosis is promoted [81]. The rutin-vanadium compound efficiently caused cell death by interfering with Bax, p53, and Bcl-2 and lowering VEGF expression in cancer cells [44].

Furthermore, rutin has also been demonstrated to suppress VEGF expression and angiogenesis in MDA-MB-231 carcinoma cells [82]. In the colon cancer model, rutin exerted the most potent cytotoxic activity on SW480 cells. These effects declined with increasing doses. In comparison to untreated animals, treatment with 20 mg/kg rutin reduced VEGF serum levels by 55%, extended lifespan by 50 days, and showed no adverse effects on body weight (BW) or relative organ weight in mice [83].

By decreasing the levels of VEGF and transforming growth factor (TGF)-β1, rutin inhibited angiogenesis of GL-15 cells [84]. In breast cancer (MCF-7 and MDA-MB-231) cell lines, rutin modulated proliferation, metastasis, and angiogenesis. Additionally, in MDA-MB-231 and MCF-7 cells, rutin modulated angiogenesis and metastasis-related genes by downregulating THBS1 and CDH1 genes, and upregulating FN1, MKI67, CDH2, VIM, and VEGFA genes [85]. In conclusion, rutin exerts anti-angiogenic effects by attenuating VEGF signaling and associated pro-angiogenic factors, thereby inhibiting tumor vascularization and progression.

### 3.8 Suppression of AP-1 transcription factor

AP-1 is a transcription factor complex involved in the modulation of gene expression linked to proliferation, angiogenesis, and survival. Its dysregulation drives tumor progression by promoting the expression of cancer-related genes that trigger mitogenic, pro-angiogenic, and anti-apoptotic signaling pathways [86,87,88,89]. Natural products or their active molecules have been shown to exert anticancer effects by inhibiting AP-1. A study on anti-metastatic actions of *Phyllanthus urinaria* L extracts (PUE) provided evidence suggesting that several polyphenols, including methyl gallate, gallic acid, epicatechin, gallocatechin-3-gallate, rutin, and naringin, contribute to the anticancer effects of this plant. PUE significantly inhibited the migration and invasion of metastatic A549 and Lewis lung cancer cells. Furthermore, PUE demonstrated the ability to block NF-κB and AP-1 nuclear translocation as well as their DNA-binding activity [90].

A different study using the human promyelocytic leukemia (HL-60) cell line found that cotreatment with rutin and vitamin E (VE) significantly decreased the expression of the c-Jun protein and diminished the binding affinity of nuclear factor-activator protein-1 (AP-1) to the VEGF gene promoter. This is accompanied by reduced VEGF expression. This inhibitory potential was attributed to downregulation of insulin-like growth factor 1 receptor (IGFR) and IRS-1, resulting in reduced AP-1 transcriptional activity [80].

Collectively, rutin’s suppression of AP-1 contributes to its anticancer activity by inhibiting the expression of key oncogenic mediators.

### 3.9 Modulation of autophagy

Autophagy was first introduced by Christian de Duve in 1963 as a lysosome-mediated disposal process [91]. Autophagy is a lysosome-dependent degradation process that maintains cellular homeostasis. Autophagy is an intracellularly regulated process necessary for preserving cellular homeostasis by eliminating misfolded and undesirable proteins [92]. Autophagy has both tumor-promoting and tumor-suppressive properties, making it a two-edged sword [93]. Autophagy controls pro-survival or pro-death signaling pathways in many cancer types and is impacted by environmental factors such as stress and the kind and stage of malignancies [94]. Rutin modulates autophagy, which contributes to its involvement in cancer control. A recent study investigated the function of rutin in hepatocellular carcinoma (HCC) autophagy and chemoresistance produced by sorafenib (SO). The results showed that rutin treatment decreases the number of autophagosomes formed following rutin-treated HCCLM3/SO and HepG2/SO cells, as well as BRAF-activated non-protein coding RNA (BANCR) expression and autophagy in SO-resistant cells. Furthermore, rutin inhibits autophagy via the BANCR/miRNA-590-5P/OLR1 axis, according to an *in vivo* investigation [95]. BANCR, miRNA-590-5p, and OLR1 are molecules involved in various cellular processes, particularly in cancer and other diseases. BANCR is a long non-coding RNA, while miRNA-590-5p is a microRNA, and OLR1 encodes for the protein LOX-1 (Lectin-like oxidized low-density lipoprotein receptor 1). HCCLM3/SO and HepG2/SO cells are derived from HCC.

Park et al. showed that rutin-activated autophagy in lung (A549), oral (CA9-22), and leukemia (THP-1) cell lines. The inhibition of rutin-induced autophagy increased TNF-α, an important regulator of inflammation. Together, these results revealed that rutin increased autophagy, which decreased TNF-α production [96]. Rutin inhibits JNK-mediated autophagy in GBM multiforme, thereby increasing the therapeutic efficacy of temozolomide (TMZ) in both *in vitro* and *in vivo*. This was demonstrated by immunoblotting analysis, which showed that TMZ activated JNK signaling to induce protective autophagy [97]. Hence, modulation of autophagy by rutin may offer a strategic pathway to target the mechanisms underlying cancer cell survival and resistance to treatment.

### 3.10 Inhibition of PI3K/AKT/mTOR pathway

In eukaryotic cells, the PI3K/AKT/mTOR (PAM) signaling pathway is a highly conserved signal transduction network that supports cell growth, survival, and cell cycle progression. Phosphorylinositol 4,5 bisphosphate (PIP2) is phosphorylated by the PI3Ks to Phosphorylinositol 3,4,5-triphosphate (PIP3), leading to the activation of serine/threonine kinase. Activated Akt to become phosphorylated, affecting cancer cells’ proliferation, survival, and cycling [98]. The tumor suppressor phospholipase and tensin homolog deleted on chromosome ten (PTEN) counteracts this pathway by dephosphorylating PIP3 back to PIP2 [99].

A new study used the argyrophilic nucleolar regulatory area and the mTOR-signaling pathway to investigate the anti-tumor activity of rutin at various doses. In the mice with solid tumors, rutin (25 and 50 mg/kg) was administered intraperitoneally, while in the experimental groups, EAC cells were induced subcutaneously. Using immunohistochemistry, it was shown that there was a significant decrease in the expression of AKT, PI3K, mTOR, and F8, especially in the groups that received rutin (25 mg) as opposed to the control group. The average AgNOR number and AgNOR area/nuclear area (TAA/NA) were calculated, and statistically significant differences were found between the TAA/NA ratio groups. Significant statistical variations were seen in the mRNA expression of the mTOR, PI3K, and AKT1 genes. Rutin had anti-tumor properties overall, both *in vitro* and *in vivo*, against the growth of solid tumors produced by EAC cells [100]. Therefore, the inhibition of the PI3K/AKT/mTOR pathway by rutin represents a key molecular mechanism underlying the antitumor potential of rutin.

## 4 Role of rutin in different cancers

Rutin has been discovered to combat multiple types of malignancies. Numerous molecular processes, both *in vivo* and *in vitro*, demonstrate rutin’s involvement in cancer (Table 2). The role of rutin in different cancers is described as:

**Table 2:** Role of rutin in management of different cancers

Cancer types	Study types	Model type	Outcomes after rutin treatment	Refs
Lung cancer	*In vitro*	Human lung epithelial cancer	Inhibited proliferation, migration, and induced apoptosis	[105]
	*In vitro*	Human lung cancer cells	Induces apoptosis, enhances the expression of GSK-3β and TNF-α	[72]
	*In vivo*, C57BL/6 mice	Lung metastasis in C57BL/6 mice	The lung tumor nodule formation inhibited, and life span was increased	[107]
	*In vitro*, A549	Human lung cancer cells	Induced autophagy, inhibited the production of TNFα, and inhibited NF-κB signaling	[96]
Brain cancer	*In vitro*, GL-15	Human GBM cell line	Induced apoptosis, delayed cell migration and upregulated the expression of metalloproteinase	[109]
	*In vitro*, GL-15	Human GBM cell line	Reduced the viability of cancer cells, downregulated the expression of miRNA-125b and STAT3	[32]
	*In vitro*, CHME	Human glioma cells	Induced cytotoxicity and apoptosis via P53 upregulation; promoted nuclear condensation, membrane blebbing, and fragmentation was caused by treatment	[41]
	*In vitro* (D54-MG, U87-MG and U251-MG) & *in vivo* (BALB/c athymic mice)	Human GBM cell line & male nude mice tumor	Rutin with TMZ enhanced the cytotoxicity; cotreatment reduced tumor volumes; reduced autophagy	[97]
Breast cancer	*In vitro*, MDA-MB-231& MCF-7	Breast cancer cells	Modulated the proliferation of cancer and the migration of the cells; Downregulated THBS1 and CDH1 genes and upregulated FN1, MKI67, CDH2, VIM, and VEGFA in cancer cells	[85]
	*In vivo*, female albino rats	Breast carcinogenesis induced by DMBA	Ameliorated the carcinogenic effect and liver function enzymes; Increased antioxidant enzymes and reduced the inflammatory markers	[30]
	*In vitro*, 4T1	Breast cancer cell line	Suppressed cell proliferation; Increased the expression of miR‐129‐1‐3p; Confined the growth of mouse breast cancer cells	[115]
Cervix cancer	*In vitro*, SiHa	Cervical cancer cells	Modulated tumor suppressor genes and oncogenes; Induced apoptosis and activation of Caspase	[119]
	*In vitro*, C33A	Human cervical cancer	Induced ROS production, reduced cell viability and nuclear condensation, provoked apoptosis by activating caspase-3 and reducing MMP	[67]
Ovarian cancer	*In vitro*, OVCAR-3	Human ovarian cancer cells	Exhibited different levels of inhibition in VEGF expression	[124]
Prostate cancer	*In vitro*, PC3	Prostate cancer cell line	5-FU/rutin combination enhanced apoptosis and p53 gene expression, downregulated Bcl-2 protein	[42]
Colon cancer	*In vitro*, HT-29	Colon cancer cell line	Showed anticancer effects, induced apoptosis, regulated the expressions of genes linked to apoptosis and inflammation	[142]
	*In vitro*, HT-29	Human colon adenocarcinoma cells	Reduced cell proliferation and increased apoptotis, modulated MMP in the group treated with Gy and rutin.	[144]
	*In vivo*, rat model	Colon cancer model induced by azoxymethane	Reduced the number of aberrant crypt foci, promoted apoptosis	[150]
Liver cancer	*In vivo*, male wistar rats	HCC	Decreased liver weight and tumor marker enzymes and liver marker enzymes	[156]
	*In vivo*, male albino rats	HCC induced by chemicals	Decreased enzyme activities, decreased VEGF levels Rutin improved hepatocellular architecture with minor hepatic changes	[110]
	*In vitro*, HepG2	Human hepatoma cells	Decreased ROS and MDA concentration	[158]
Gastric cancer	*In vitro*, SGC-7901 cells	Human gastric adenocarcinoma	Inhibited the growth of cancer cells, and induced cell cycle arrest at G0/G1, increased the expression of Caspase, reduced the Bcl-2/Bax ratio	[163]
	*In vitro*, AGS and MGC803	Gastric cancer cells	Inhibited the proliferation, migration, as well as invasion, induced apoptosis, and suppressed the EMT process	[164]
Leukemia	*In vitro*, U937, KG1, HL-60 and KG1a	Human leukemic cells	Induced apoptosis in adherent leukemic cells, inhibitory effect on the survival of adherent leukemic progenitors	[170]
	*In vivo*, BALB/c mice	Animal model leukemia	Decreased the percentage of Mac-3 markers, reduced weights of the spleen and liver	[171]
	*In vitro*, HL-60 cells	Human promyelocytic leukemia	VEGF expression attenuated by rutin with vitamin E	[80]
Bone cancer	*In vitro*, SAOS2	Osteosarcoma cell lines	Reduced the proliferation, migration, of cancer cells and induced apoptosis	[179]
Skin cancer	*In vitro*, RPMI-7951 and SK-MEL-28	Human melanoma cell lines	Reduced the viability, exerted a senescence-inducing potential	[183]
	*In vitro*, B164A5	Murine melanoma cell line	It demonstrated to be an active pro-apoptotic and antiproliferative compound against B164A5 cells	[186]
Renal cancer	*In vitro*, 786-O	Renal cancer cell	Reduced the cell viability, induced a substantial increase in sub-G1 population, decreased G0/G1 population	[190]
Pancreatic cancer	*In vitro*, PANC-1	Human pancreatic cancer cell line	Reduced growth in a dose-dependent manner, upregulated miRNA-877-3p expression	[66]
Multiple myeloma	*In vitro*, RPMI8226	Multiple myeloma cells	Demonstrated antioxidant action and cytotoxicity	[202]
	*In vitro*, ARH-77	Multiple myeloma cells	Demonstrated cytotoxic effects	[203]

### 4.1 Lung cancer

Lung cancer is one of the leading causes of cancer-related morbidity and mortality rates worldwide, with non-small cell lung cancer (NSCLC) accounting for over 85% of all cases [101]. Currently, the primary clinical treatments for NSCLC are surgical resection, chemotherapy, and radiotherapy. For early-stage NSCLC, timely surgery can lead to a curative outcome [102,103]. Although the significance of immunotherapy in treating NSCLC has grown in recent years, the response rate remains below 40% in specific populations [104]. Rutin-loaded liquid crystalline nanoparticles (LCNs) exhibited promising anti-proliferative and anti-migratory properties [105]. An experiment was performed to evaluate the expression of NF-κB and p38 under the intervention of rutin in lung cancer. It was reported that treatment with rutin as well as cisplatin led to an increase in the subpopulation of sub-G1 phase cells and a decrease in the cells in the G2 phase, indicating its ability to regulate the growth of cells. Furthermore, rutin reduced the expression of P38 and NF-κB, suggesting that rutin exerts its anticancer effects through modulation of signaling pathways [106]. The genoprotective effects of β-carotene and different flavonoids (naringin, quercetin, or rutin), both individually and in combination, were explored against NNK-induced DNA damage. Human lung cancer cells were pre-treated with β-carotene, a flavonoid, or a combination of both for 1 h before being exposed to NNK for 4 h. The study found that β-carotene increased NNK-induced DNA strand breaks and 7-mGua levels. In contrast, when cells were treated with naringin, quercetin, or rutin, there was a significant reduction in NNK-induced DNA strand breaks, with quercetin showing the most substantial effect, followed by naringin and rutin [107]. The rutin and cisplatin groups exhibited significantly higher TNF-α expression than the control group. Furthermore, DAPI staining indicated more apoptotic cells in the rutin and cisplatin groups than in the control group. Immunofluorescence analysis revealed that GSK-3β expression was markedly higher in the cisplatin and rutin groups compared to the control group. In conclusion, rutin enhances TNF-α-induced apoptosis of A549 human lung carcinoma cells [72]. When administered orally at a concentration of 200 nmol/kg BW, rutin markedly inhibited lung tumor nodules by 63.59% and extended the life span [108]. A study was made to analyze the effect of rutin on autophagy and inflammation in cancer cell lines. Remarkably, rutin induced autophagy in lung, leukemia, and oral cancer cell lines, which is linked with suppressed TNF-α production [96]. In summary, rutin exhibits significant anticancer potential against lung cancer by inducing apoptosis, inhibiting NF-kB and p38 signaling, reducing DNA damage, suppressing inflammation, promoting autophagy, and enhancing the efficacy of chemotherapeutic drugs.

### 4.2 Brain cancer

Flavonoids, including rutin, have demonstrated significant anticancer effects in GBM cell models by reducing mitochondrial metabolism and cell viability, inducing apoptosis through damage to the rough endoplasmic reticulum and mitochondria. Flavonoids also caused a delay in and impaired cell migration via downregulation of metalloproteinase (MMP-2) and a reduction in filopodia-like structures on the cell surface. It further enhanced intracellular expression of laminin and intra-and extracellular expression of fibronectin [109]. The use of rutin in cancer management, including brain cancer, has been shown to play a significant role in the modulation of various cell signaling molecules.

Another study demonstrated that rutin reduced the viability of GL15 cells and the expression of miRNA-125b, underscoring the flavonoid’s anti-glioma properties. This highlights its potential as a promising adjunctive therapy for GBM [32]. In CHME cells, rutin-induced cytotoxicity was linked to apoptosis via p53 upregulation, evidenced by nuclear condensation, membrane blebbing, and fragmentation, as evidenced by DAPI staining. The release of cytochrome c further confirmed the apoptotic effect, the activation of caspase 9 and caspase 3, upregulation of BAX, and downregulation of BCL expression [41].

A study explored the synergistic effects of rutin and TMZ in GBM multiforme. The *in vitro* cell viability assay showed that rutin by itself generally had a low cytotoxic effect, but it did enhance the effectiveness of TMZ in a manner that depended on the dosage. Subcutaneous and orthotopic xenograft studies further indicated that mice receiving combined TMZ/Rutin treatment exhibited significantly lower tumor volumes than those treated with TMZ or rutin alone. Additionally, immunoblotting analysis revealed that TMZ stimulated JNK activity to trigger a protective autophagic response, which rutin inhibited, leading to reduced autophagy and increased apoptosis [97]. Treatment with rutin at 50–100 μM concentrations resulted in decreased proliferation and viability of GL-15 cells. This was accompanied by a reduction in ERK1/2 phosphorylation (P-ERK1/2) levels and an accumulation of cells in the G2 phase of the cell cycle. Additionally, exposure to 100 μM rutin led to apoptosis in 87.4% of GL-15 cells [73].

### 4.3 Breast cancer

**Figure 3:**
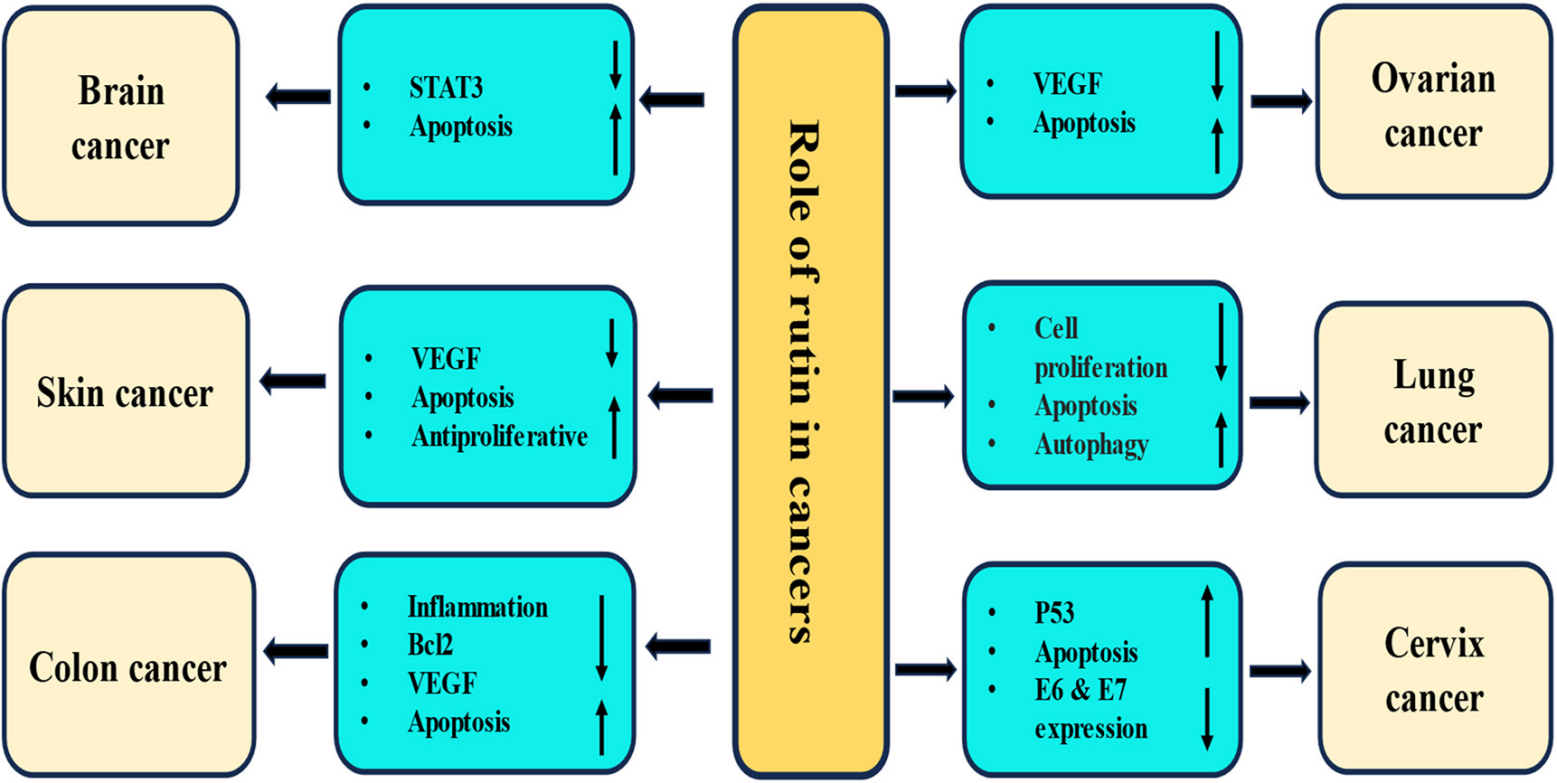
Rutin exerts its anticancer effects through inhibition of inflammation, cell proliferation, angiogenesis, signal transducer and activator of transcription, induction of apoptosis, and activation of tumor suppressor genes. The downward-pointing arrow shows downregulation, whereas the upward arrow represents upregulation.

Breast cancer is the most common form of cancer in females, with around 2.3 million new cases diagnosed each year, accounting for 11.7% of all cancer diagnoses [110,111]. Globally, breast cancer represents 25% of cancer cases and 16.7% of cancer-related deaths in most countries [111]. The current mode of treatment for this pathogenesis is effective, but it also causes various side effects. Clinicians need alternative therapies that can effectively cure breast cancer without adverse effects, so it is essential to supplement current chemotherapeutic drugs with drugs based on natural compounds/medicinal plants. In this regard, previous studies have demonstrated the anticancer potential of natural compounds in the protection and management of breast cancer [112]. Rutin in breast cancer prevention is explained through modulation of various cell signaling pathways (Figure 3).

The use of rutin in cancer management, including breast cancer, has shown a significant role in the modulation of various cell signaling pathways. A recent study evaluated the role of rutin on the proliferation, angiogenesis, and metastasis of breast cancer cell lines. Dose-dependent treatment with rutin upregulated genes, including FN1, MKI67, CDH2, VIM, and VEGFA, while downregulating THBS1 and CDH1 genes [85].

A nanohybrid formulation of rutin was developed by loading it onto 4-carboxyphenylboronic acid (PBA) linked and amine-functionalized MgO nanoparticles (MgO-NH-PBA). This MgO-NH-PBA-rutin nanohybrid showed significant anticancer activity against MDA-MB-231 cells through intracellular ROS-mediated apoptosis and inhibited the migration of cancer cells [113]. The growth of the TNBC MDA-MB-231/GFP orthotopic xenograft in the nude mice model was considerably inhibited by an intraperitoneal injection of rutin at a dose of 30 mg/kg, three times per week. These findings unequivocally identify rutin, a functional dietary flavonoid, as a promising treatment and preventative option for c-Met-dependent breast cancers [114].

Rutin suppresses cell proliferation and increases the expression of miR‐129‐1‐3p in 4T1 cells. miR‐129‐1‐3p overexpression inhibits the proliferation, invasion, migration, and calcium overload of mouse breast cancer cells and increases apoptosis. Knockdown of miR‐129‐1‐3p reversed these effects, suggesting that rutin exerts its anticancer effects against mouse breast cancer cells through regulation of miR‐129‐1‐3p/Ca2+ signaling pathway [115]. Glycosylated flavonoids such as rutin (found in cranberries) and naringin (a constituent of citrus fruits) demonstrated potent anticancer activity, even at the lowest concentration in human breast cancer cells [82].

### 4.4 Cervical cancer

Cervical cancer is one of the most common gynecological malignancies globally, ranking fourth among female malignancies and even taking the top spot in some developing countries [116]. The 5-year survival rates by histologic type are 75.8% for adenocarcinoma and 81.4% for squamous cell carcinoma, with adenocarcinoma having a worse prognosis than squamous cell carcinoma [117]. Chemotherapy, surgery, and radiotherapy are treatment modalities for cervical cancer. However, these treatments also cause side effects. Therefore, it is essential to explore natural compound-based therapies to mitigate these complications [118].

Furthermore, the FITC-Annexin V/PI double staining method confirmed the induction of apoptosis [119]. The anticancer effect of rutin was explored using key biomarkers, including nuclear condensation and apoptosis, ROS, and changes in MMP. It was reported that rutin treatment reduced cell viability and induced a dose-dependent increase in ROS production and nuclear condensation [67]. Rutin treatment showed decreased cell viability with increased cell accumulation in the G0/G1 cell cycle phase in HeLa cell lines. Moreover, rutin treatment has led to the downregulation of E6 and E7 expression linked with increased p53 and pRB levels [120].

By causing apoptosis, G0/G1 phase arrest, and downregulating the levels of Notch-1 and Hes-1 of the Notch signaling pathway, rutin demonstrated strong anticancer effects in human cervical carcinoma Caski cells [121]. In the G0/G1 phase of the cell cycle, HeLa cell lines treated with rutin showed decreased cell viability and increased cell accumulation. Furthermore, rutin therapy has been linked to an increase in p53 and pRB levels, accompanied by a downregulation of E6 and E7 expression. This has also led to increased expression of Bax and decreased expression of Bcl-2, which releases cytochrome c into the cytoplasm and activates the caspase cascade by cleaving caspase-3, caspase-8, and caspase-9. Thus, by focusing on two important oncoproteins linked to viral progression, rutin is suggested as one of the most effective medication candidates for treating cervical cancer [120]. Rutin was documented to inhibit the growth of Caski cervical cancer cells in a dose-dependent manner. Through the activation of caspase-3/9, the production of ROS, and changes in the expression of Bax/Bcl2 mRNA, rutin caused notable apoptotic effects. Rutin administration reduces the expression of Notch-1 and Hes-1 mRNA. Through inducing apoptosis, causing G0/G1 phase arrest, and downregulating the levels of Notch-1 and Hes-1 in the Notch signaling pathway, these findings collectively demonstrate that rutin has strong anticancer effects in human cervical carcinoma Caski cells [67]. An innovative phytotherapy for cervical cancer based on the rutin–fucoidan complex was studied in order to increase bioavailability and induce cancer cell death. A novel flavonoid-polysaccharide combination called rutin–fucoidan (Ru–Fu) was created. In HeLa cells, the Ru–Fu combination effectively contributes to anti-proliferation, S-phase cell cycle arrest, and the induction of cell death associated with apoptosis [122].

### 4.5 Ovarian cancer

Rutin-loaded PCL-PEG was made, and its anti-cancer effects against human ovarian cancer were tested. Cytotoxicity testing demonstrated that RUT-loaded PCL-PEG improved cytotoxicity in a dose- and time-dependent way. In treated MDA-MB-231 cells with RUT-loaded PCL-PEG, a significant upregulation of Bax genes and caspase-8, caspase-9, and caspase-3 was observed compared to cells treated with free rutin [123]. The results of another study revealed that all the tested flavonoids exhibited different levels of inhibition in VEGF expression. The inhibitory strength of VEGF protein secretion was found to be in the following order: genistein > kaempferol > apigenin > quercetin > tocopherol > luteolin > cisplatin > rutin > naringin > taxifolin [124]. Ru-NLC has a significant effect on SK-OV-3 cell metabolism, which in turn increases the levels of apoptosis-related proteins. The cell’s ability to proliferate is reduced as a result of these changes, and intrinsic apoptosis finally causes it to die. The Western blotting experiment’s findings indicate that Ru-NLC induces cancer cells to undergo increased apoptosis [125]. Another study showed that rutin could directly stimulate ovarian cell steroidogenesis while also inhibiting the cells’ survival, proliferation, and apoptosis, as well as their response to FSH’s stimulatory activity. It is essential to consider the potential adverse effects of rutin on ovarian cells when consuming foods and using medications that contain rutin and its metabolites [126]. Rutin’s protective effect against acrylamide-induced ovarian inflammation, oxidative stress, DNA damage, and hormonal changes was assessed. The findings demonstrated that RU provided significant protection against oxidative stress, DNA damage, and inflammation induced by AA, likely due to its antioxidant properties [127].

### 4.6 Prostate cancer

Prostate cancer stands as the second most prevalent cancer in men and bears the fourth highest mortality rate among men worldwide. The anticipated surge in prostate cancer cases from 1.41 million in 2020 to 2.43 million in 2040 underscores its substantial socioeconomic burden [128]. The current mode of treatment for prostate cancer is expensive and also shows adverse effects. In this regard, natural products have proven their role in prostate cancer management. Evidence from the literature suggests that various natural products can selectively target multiple molecules and signaling pathways associated with the development and advancement of tumors [129,130,131,132,133]. The use of rutin in cancer management, including prostate cancer, has been shown to play a significant role in modulating various cell signaling molecules. The study examined the combined effects of rutin with the widely used anticancer drug, 5-fluorouracil (5-FU), on the prostate cancer cell line (PC3). When the cells were treated separately with rutin and 5-FU, the 50% inhibitory concentrations (IC_50_) were reported as 900 μM and 3 mM, respectively. However, the combination index of the combined 5-FU/rutin application indicated synergistic effects [42].

An extensive in-silico investigation into rutin compounds as potential inhibitors of prostate cancer signaling pathways revealed that the AKT, EGFR, and ERK signaling pathways were targeted by rutin derivatives, which could prevent prostate cancer from spreading [134]. WST-1, Annexin V ELISA, DAPI, and Acridine Orange staining were used to assess the anticancer efficacy of rutin in prostate cancer cells (PC-3), and the Scratch Assay test was used to assess rutin’s anticancer and anti-metastatic qualities. It has been established that rutin can both promote apoptosis and prevent the epithelial–mesenchymal transition [135]. In rats, rutin naturally protected the prostate cells from damage caused by zinc nanoparticles [136]. Rutin can increase the effectiveness of chemotherapy and is a potent senomorphic drug that targets senescent cells. The malignant phenotypes of prostate cancer cells, such as *in vitro* proliferation, migration, invasion, and most importantly chemoresistance, were improved by conditioned media generated by senescent stromal cells; however, rutin markedly inhibited these gain-of-function effects. Combining a chemotherapeutic medication with rutin significantly improved the treatment success, even when traditional chemotherapy decreased tumor development [137].

### 4.7 Colon cancer

Colorectal cancer remains the primary cause of mortality, with the rise in its occurrence in Western countries possibly linked to lifestyle changes. Specifically, alterations in dietary patterns could account for this trend [138,139]. About one-third of metastatic colorectal cancer (mCRC) patients have localized liver metastases and may be eligible for potentially curative surgery following systemic therapy [140]. Regrettably, despite the progress in existing treatments, the 5-year survival rate for metastatic CRC patients remains low, at less than 15% [141]. Consequently, there is a clear need for new strategies and agents to enhance the prevention and treatment of CRC. Rutin, as well as Silibinin, exhibited anticancer potential through the induction of apoptosis and the regulation of gene expressions associated with apoptosis, inflammation, and mitogen-activated protein kinase (MAPK) pathway proteins more efficiently in combination than separately [142]. Treating colon cancer cells HT-29 with hyperoside and rutin decreased cell viability dose dependently. The simultaneous activation of the mitochondria-dependent apoptotic pathway by hyperoside and rutin occurred via modulation of B-cell lymphoma 2 (Bcl-2) expression and Bcl-2-associated X protein (Bax), resulting in the activation of cleaved caspases-3, -8, and -9, as well as cleaved poly-(ADP-ribose) polymerase [143].

The cytotoxicity study revealed that exposing colon cancer HT-29 cells to varying concentrations of rutin resulted in a dose- and time-dependent decrease in cell proliferation. Furthermore, after irradiation, it was noted that the combined impact of 4 Gy radiation and rutin at a concentration of 80 µM resulted in reduced cell viability compared to cells treated with rutin alone and those exposed to 4 Gy radiation alone [144]. Diets containing quercetin, curcumin, silymarin, ginseng, and rutin decreased the number of aberrant crypt foci compared to the control group. Analysis of the colon mucosa revealed that all the herbal supplements, except silymarin, stimulated apoptosis, with quercetin demonstrating the most potent effect [145]. Rutin exhibited the most significant cytotoxic effects against SW480 cells compared to other cancer cell lines. It also reduced the tumor volume, and it increased the mean survival time by 50 days, while lowering VEGF serum levels by 55% compared to untreated mice [83]. By triggering apoptosis through cell cycle arrest and caspase protein activation, rutin prevents colon cancer cells from proliferating. Furthermore, rutin administration markedly increased the expression of β-actin and caspase-3 in HCT116 [146]. To ascertain rutin’s effectiveness in treating A549 lung cancer cells and colon cancer cells, the cytotoxic effect of rutin in the preemulsion was examined using *in vitro* tests. The findings showed that rutin in the prenanoemulsion effectively inhibited colon and lung cancer cells [147]. The growth of Caco-2 cells is inhibited more effectively by rutin and anticancer drugs (5-FU and/or oxaliplatin) when taken together than when taken separately. Lower dosages of 5-FU and oxaliplatin, which have comparable efficacy, may also be helpful in minimizing any potential side effects of these drugs [148]. The *in vitro* cytotoxicity of rutin, rutin-PVP K30 SD, frankincense, and a combination of rutin-PVP K30 SD and frankincense was evaluated against human colon cancer (HCT-116) cell lines. The combination of frankincense and rutin-PVP K30 SD was more successful than either frankincense or rutin alone at stopping the growth of cancer cells [149]. CF1 female mice were given dietary quercetin and rutin for 50 weeks in order to evaluate their capacity to prevent colonic neoplasia caused by azoxymethanol (AOM). Quercetin and rutin show notable efficacy in lowering the incidence of FAD and the hyperproliferation of colonic epithelial cells brought on by AOM [150].

### 4.8 Liver cancer

The world’s sixth most frequently diagnosed cancer is primary liver cancer, with HCC being the most common histological type [111,151]. Chromosomal alterations, genetic mutations, hormonal changes, and changes in signaling pathways are the chief players in this pathogenesis [152,153]. Effective therapeutic options are available for patients with HCC, significantly improving survival rates. Nevertheless, a considerable portion of patients do not have a positive response to locoregional or systemic treatment and ultimately succumb to their illness [154,155]. The use of rutin in cancer management, including liver cancer, has been shown to play a significant role in modulating various cell signaling molecules.

Zhou et al. experimented to explore the molecular mechanism of rutin in SO-induced autophagy and chemoresistance in HCC. Results indicated that rutin treatment reduces autophagy and BANCR expression in SO-resistant cells. Transmission electron microscopy revealed a significantly reduced number of autophagosomes in rutin-treated HepG2/SO and HCCLM3/SO cells [95]. A study was conducted to examine the effects of rutin on liver carcinoma. Results reported a substantial increase in relative liver weight and tumor marker enzymes; liver marker enzymes and abnormalities were noticed in membrane-bound enzymes as well as electrolytes, while such alterations were meaningfully restored in the rutin-treated Group as compared to the treated Group [156].

A study evaluated the potential of chitosan/poly (acrylic acid) nanogel (CAN) in enhancing the anticancer effects of rutin. The formulation was tested for its ability to combat liver cancer in rats. The results showed that levels of liver function enzymes and total bilirubin were significantly higher in the Group with liver tumors induced by DENA/carbon tetrachloride (CCl_4_) as compared to the control group. In the HCC Group, VEGF levels were higher than in the control Group. However, rutin administration notably decreased VEGF levels in both the free and loaded Groups compared to the HCC group. The serum AFP level also increased in the HCC group compared to the control Group, indicating tumor cell proliferation and HCC progression in the DENA/CCl_4_-treated rats. However, in the rutin Group, histopathological examination of the liver sections revealed improved hepatocellular architecture with minor hepatic changes such as hepatocyte swelling and vacuolations [110]. The research aimed to create poly(lactic-co-glycolic acid) (PLGA) nanoparticles (NPs) containing rutin (RT) for treating HCC. RT-PLGA-NPs demonstrated considerable enhancements in hematologic, renal biochemical, and hepatic parameters when evaluated preclinically for anticancer effects through oral administration. Particularly notable improvements were observed in the regulation of inflammatory markers, oxidative stress, cytokines, and antioxidant enzymes associated with liver tissue damage [108]. The role of rutin isolated from the *Morinda citrifolia* plant against hepatic carcinoma (HepG2) cell lines was explored. It was reported that cell viability was decreased with an increase in the concentration of rutin [157]. Another study sought to determine if quercetin and rutin could have chemoprotective effects on the growth, viability, and antioxidant defense system response of a human hepatoma cell line (HepG2). The findings show that both natural antioxidants cause beneficial alterations in the cultured HepG2’s antioxidant defense system, which stop or postpone circumstances that encourage cellular oxidative stress [158].

### 4.9 Gastric cancer

Gastric cancer (GC) ranks among the top causes of cancer-related mortality, and its incidence exhibits significant geographic variability [159]. Gastric cancer remains an important global health concern with a dismal prognosis. A considerable number of patients are diagnosed with advanced-stage gastric cancer, leading to minimal life spans due to delayed diagnosis [160]. Patients with metastatic disease typically have a survival of just over 1 year, even with the use of targeted therapy [161,162]. Rutin has been found to inhibit the growth of human gastric adenocarcinoma SGC-7901 cells, leading to cell cycle arrest at the G0/G1 phase, a shift in the Bcl-2/Bax ratio, and an increase in the expression of caspase-3, caspase-7, and caspase-9. When rutin is administered in combination with oxaliplatin, it demonstrates a synergistic anticancer impact, enabling a reduction in the oxaliplatin dosage and subsequently reducing its toxicity [163].

In 2024, Huang et al. reported that rutin significantly inhibited the proliferation, migration, and invasion abilities of gastric cancer cells, inducing apoptosis. Further experiments proved that rutin achieved this effect by suppressing the activation of the Wnt/β-catenin pathway, thereby inhibiting the biological behavior of these cancer cells [164]. Whey protein isolate WPI and rutin together dramatically increased *in vitro* cell migration in L929 monolayers, suggesting that they can heal wounds and treat stomach ulcers [165]. Furthermore, the apoptotic processes of rutin were linked to caspase-mediated signaling; however, the combination of rutin with the chemotherapeutic drug Oxaliplatin increased the death of gastric cancer cells SGC-7901, as evidenced by a lowered BCL-2/Bax ratio [163]. The protective effect of rutin against acute gastric mucosal lesions caused by ischemia-reperfusion (I/R) was investigated in a study. Rutin at doses of 50, 100, and 200 mg/kg significantly inhibited the rise in the stomach mucosal injury index induced by gastric I/R compared to the I/R group [166].

Rutin significantly increased the rates of apoptosis in AGS and MGC803 cells. This suggests that Rutin may partially mediate its antiproliferative effect on gastric cancer cells by inducing apoptosis [164].

**3:** Rutin exerts its anticancer effects through inhibition of inflammation, cell proliferation, angiogenesis, signal transducer and activator of transcription, induction of apoptosis, and activation of tumor suppressor genes. The downward-pointing arrow shows downregulation, whereas the upward arrow represents upregulation.

### 4.10 Leukemia

Leukemia is a malignant disorder characterized by the uncontrolled clonal proliferation of leukocytes in the bone marrow and peripheral blood [167,168]. In 2020, the World Health Organization (WHO) reported 474,519 new cases of leukemia worldwide, representing 2.5% of all cancer cases, and 311,594 new leukemia deaths, accounting for 3.1% of all malignancy deaths [111]. The current mode of treatment is causing several side effects. A recent study reported that the natural compound plays a role in anti-leukemic activity [169]. However, a safe and non-toxic treatment with no side effects is required to control this disease and overcome the adverse effects of chemotherapeutic drugs.

The flavonoidic fraction of *Hammada scoparia*, as well as its compound rutin, has been shown to induce apoptosis in adherent leukemic cells and eliminate CAM-DR. Additionally, rutin has demonstrated an inhibitory effect on the survival of adherent leukemic progenitors (CD34^+^ 38^−^123^+^). The pro-apoptotic actions of rutin have been associated with a reduction in active GSK3β [170]. The effects of rutin on WEHI-3 in BALB/c mice *in vivo* were also studied, and the outcomes indicated that rutin decreased the percentage of Mac-3 markers. The weights of the spleen and liver were reduced in rutin-treated groups, and the results showed that rutin reduced the weight of these organs [171]. After receiving a dose of 120 mg/kg of rutin, the tumors in mice were smaller and lighter than those in the control group. The tumors in the mice treated with 120 mg/kg of rutin were significantly smaller than those in the untreated control group [172]. Additionally, rutin, in combination with vitamin E, was found to attenuate VEGF expression in HL-60 cells [80]. In a combination study, the antiproliferative action of AraC was synergistically improved by isorhamnetin, kaempferol, and myricetin when combined with AraC. On the other hand, rutin and AraC were antagonistic (CIs > 1). According to the cell cycle study, the synergism of flavonoids (10 µM) with AraC (0.5 µM) results from notable alterations in the L1210 cells’ cell cycle profile. Cells in the G2/M stages were blocked when treated with a flavonoid and AraC combination [173]. A work demonstrated ZnO-Rutin NPs’ effective antitumor potential against CML cells. The proportion of early apoptosis rose marginally after exposure to ZnO-Rutin NPs, while the percentage of late apoptosis increased significantly. The downregulation of the Bcl-2 gene highlighted the mechanism by which ZnO-Rutin NPs promote apoptosis, resulting in a 0.33-fold reduction and a 1.98-fold elevation of the Bax mRNA level [174].

### 4.11 Bone cancer

Osteosarcoma, a type of bone cancer, primarily affects children and teenagers aged 10 to 14, accounting for 3–5% of childhood cancers [175,176]. Osteosarcoma has an incidence of approximately 3.5 cases per million worldwide [177]. A combination of surgery and adjuvant chemotherapy is currently the standard therapeutic approach for patients with osteosarcoma [178]. Soosai et al. experimented to explore the seeds of buckwheat (Fagopyrum sp.) for the isolation of rutin and its anticancer properties against Osteosarcoma. The study findings reported that SAOS2 cells exposed to standard rutin at 10 g/mL and rutin fraction at 20 g/mL demonstrated substantial cell shrinkage, decreased cell density, and morphological alterations, which indicate apoptotic cells. Rutin-fraction significantly increased the expression of pro-apoptotic protein Bad, resulting in the negative regulation of Bcl-2’s expression [179]. ALP activity, Runx2 protein expression, and osteoblast MC3T3-E1 development were all shown to be impacted by rutin. Alizarin red staining results demonstrated that calcified nodules developed in all groups and that the area of calcified nodules in rutin-treated groups was larger than that in the control group; the larger the area, the higher the concentration. Osteoblastic differentiation can be aided by rutin, and its effectiveness increases with concentration [180]. In rats given rutin, the protein FNDC1, which contains the fibronectin type III domain, may be a novel factor related to bone metabolism that is reduced. Through the Akt/mTOR signaling pathway, rutin may control autophagy and FNCD1 levels [181]. In a study, rutin was added to human osteosarcoma MG63 and U2OS cells at 10, 20, and 40 μmol/L. Rutin treatment at 20 and 40 μmol/L significantly reduced the rate of proliferation, increased the rate of apoptosis, decreased the ability to migrate and invade, significantly downregulated the Ki67 protein, and significantly increased the Bax/Bcl-2 ratio in MG63 and U2OS cells. All of these changes were statistically significant. Additionally, rutin decreased the expression of VEGF and Ki67 in tumor tissues, increased cell death, and dramatically suppressed the development of osteosarcoma cells *in vivo* [182].

### 4.12 Melanoma

A critical study evaluates the cytotoxic effects of rutin against two different human melanoma cell lines. The outcomes show a dose-dependent reduction in the viability of both cell lines, accompanied by a visible decrease in the cell confluency and apoptotic characteristics within the cell nuclei [183]. The antimetastatic potential of the flavonoids, including rutin, was examined, and the rutin group exhibited decreases in the growth index compared with the ethanol group [184]. In an animal model, rutin has antiangiogenic effects against melanoma. Additionally, it has been found to downregulate the expression of VEGF and IL-1β. These findings suggest that rutin’s antiangiogenic activity may be due to its modulation of these cytokines as well as growth factors [185]. Rutin showed antioxidant activity, and complexation enhances this property. Further incubation with rutin (100 µM) demonstrated that it is an active pro-apoptotic and antiproliferative compound against B164A5 cells [186]. In mouse B16F1 melanoma, the leaf extract of Mallotus japonicus and its main active ingredient, rutin, inhibited the production of melanin [187]. To assess the fisetin and rutin, and their synergistic treatment, A431 and A375 cells were utilized. Following a 24-h treatment period, cell morphology was examined, and cell viability was evaluated using the MTT test. According to the findings, the combined effect of these agents is more effective than their separate treatment, resulting in a dose-dependent reduction in cell viability and an enhancement of cell dimorphologies as concentration increases [188]. The preparation and assessment of rutin-loaded solid-lipid nanoparticles (Ru-SLNs) gel for the treatment of melanoma cells was the goal of a study. A gel based on Ru-SLNs showed encouraging promise and successfully targeted melanoma cells to the skin’s epidermal layer [189].

### 4.13 Renal cancer

The potential anticancer effects of rutin against human renal cancer cell lines were evaluated. While considering its safety in Vero kidney cells, the possible anticancer effects of rutin on a human renal cancer cell line (786-O) were evaluated, along with the suitability of ionic liquids (ILs) for enhancing drug transport. In the cancer cells, a concentration-dependent reduction of cell viability was noticed. The outcomes presented that rutin induced a notable decrease in cell viability compared to non-treated control cells. Additionally, rutin (50 µM) induced a substantial increase of approximately 30% in the sub-G1 population, a decrease in the G0/G1 population, and an increase in the S population cells. At 50 µM, however, a notable reduction in cell viability was already seen in 786-O cells. Furthermore, rutin exposure led to a noteworthy rise in the sub-G1 population of 786-O cells, supporting the biomolecule’s potential anticancer properties [190].

Additionally, rutin may decrease the expression of TGF-β1, fibronectin, and collagen IV in kidney tissues. Through Western blot analysis, it was discovered that rutin could lower the expression of TGF-β1 and p-smad2 in rat kidney tissues. In 5/6-nephrectomized rats, rutin reduces renal fibrosis and proteinuria by preventing oxidative damage and blocking TGFβ1-smad signaling activation [191]. In Wistar rats, rutin inhibits cisplatin-induced kidney inflammation and death by reducing the expression of NF-κB, TNF-α, and caspase-3 [192]. In rats with obstructive nephropathy, rutin reduces kidney interstitial fibrosis. In obstructive nephropathy, rutin’s renoprotective benefits are likely attributed to its anti-inflammatory properties and suppression of TGF-β1/Smad3 signaling [193].

### 4.14 Pancreatic cancer

With over 300,000 deaths annually, pancreatic cancer ranks as the seventh most common cause of cancer-related deaths worldwide. Pancreatic ductal adenocarcinoma, an infiltrating neoplasm with glandular differentiation originating from the pancreatic ductal tree, is the most prevalent tumor form among pancreatic malignancies [194] and progresses without precise symptoms, making early diagnosis challenging. The current treatment approach for this type of malignancy involves surgery, radiotherapy, adjuvant therapy, and chemotherapy. While certain chemotherapeutic drugs and immunotherapy have been used for these patients, their effectiveness is somewhat restricted [195,196,197]. Here is a rising interest in utilizing natural compounds for chemotherapy to improve the therapeutic efficacy of specific anti-cancer medications [198].

A study on pancreatic cancer was conducted to evaluate the potent anti-proliferative, pro-apoptotic, and anti-migration potential of rutin. The results revealed that rutin effectively slowed down the growth of PANC-1 cells in a dose-dependent manner, with 5 μg/mL of rutin having a significant inhibitory effect on cell growth. Additionally, using a wound-healing assay, we observed that the migration of PANC-1 cells was markedly reduced after the administration of rutin and a decrease in the expression of the migration-related protein MMP-9. After the rutin treatment, an increase in cell apoptosis was observed compared to the untreated cells [66]. By preventing acinar to ductal metaplasia, rutin shields the pancreas from inflammatory damage and oncogene-driven carcinogenesis. It is thought that one possible way that rutin inhibits Kras-driven carcinogenesis is through AMPK activation. By blocking ADM, rutin prevented oncogenic Kras-driven carcinogenesis and AP-induced pancreatic damage [199].

In cell lines under investigation, rutin increases the quantity of caspases 3/7 and has an anti-proliferative effect. In conclusion, the normal cell lines HPDE, BxPC-3, Huh-7, MiaPaCa-2, Suit-2, and HepG-2 cells displayed the highest cellular growth inhibition. Additionally, rutin showed dose-dependent suppression of glutathione-S-transferase activity and the cytochrome P450 enzyme (CYP450 3A4). Moreover, rutin’s anti-inflammatory properties were reinforced by the inhibition of PGE2 synthesis in BxPC-3 cells (high COX-2 expression) [200].

### 4.15 Multiple myeloma

Multiple myeloma (MM) comprises nearly 10% of all blood cancers and exhibits significant variations in treatment response [201]. Natural products have been reported to play a role in the management of MM [201]. Rutin-zinc(II) has demonstrated antioxidant activity, as evidenced by its cytotoxic effects against leukemia cell lines, such as multiple myeloma (RPMI8226), *in vitro* [202]. A research study was conducted to examine the cytotoxic impact of rutin on ARH-77 cells. Following a 24-h incubation period, rutin demonstrated cytotoxic effects, particularly at concentrations of 50, 100, and 200 µM [203].

## 5 Approaches to improve the bioavailability and efficacies of rutin

It has been discovered that rutin can control cancer by modifying different cell signaling pathways. Furthermore, its function in both *in vitro* and *in vivo* cancer has been reported. Rutin’s therapeutic potential may be limited by its rapid elimination, low absorption, poor solubility, metabolism, and unstable nature, despite its numerous health benefits, particularly in cancer prevention. These factors restrict its ability to reach valuable quantities in tumor tissues. When these circumstances come together, they can greatly reduce the compound’s bioavailability, preventing the body from using it to its full potential for promoting health [204,205].

**Figure 4:**
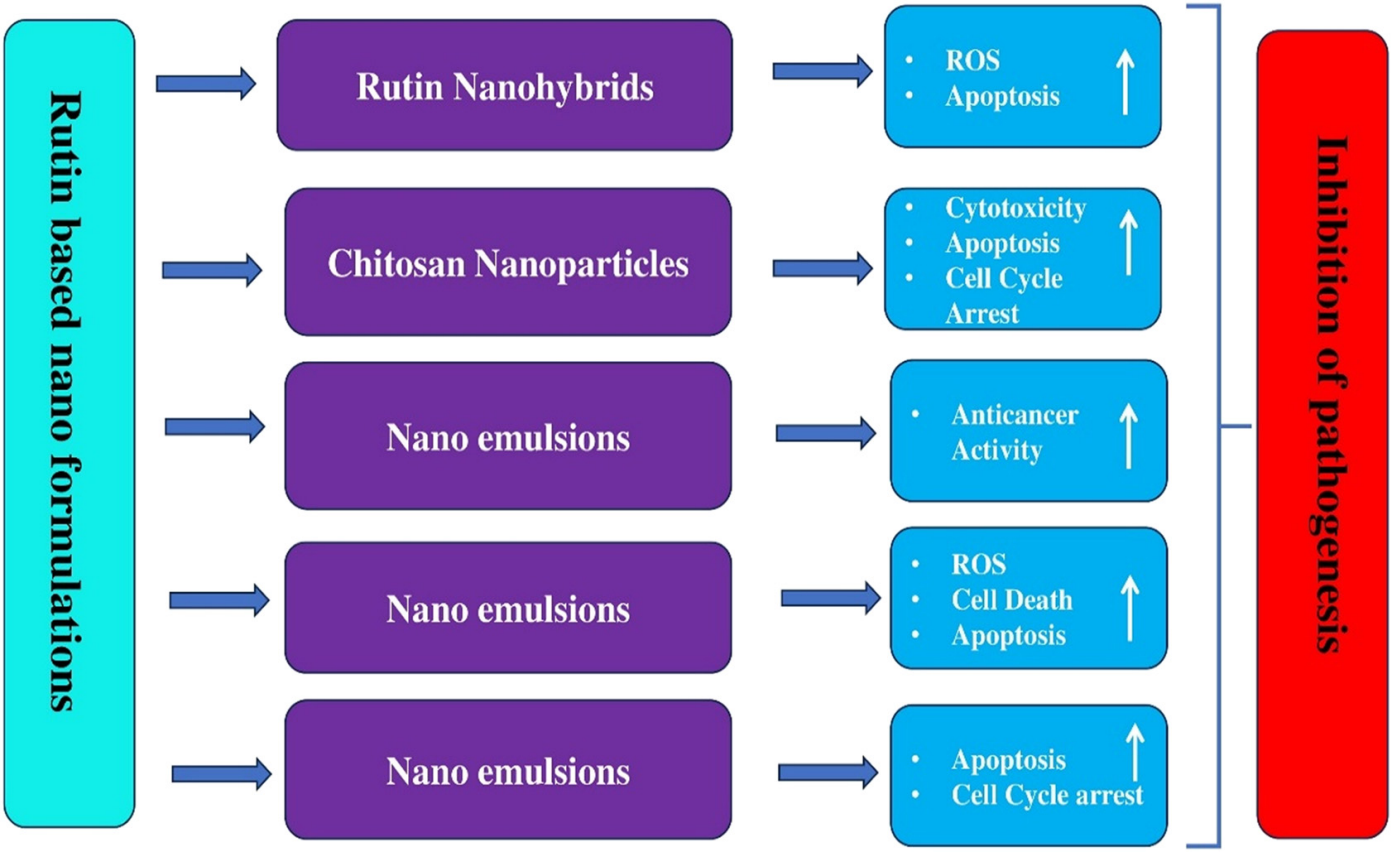
Various approaches based on nanotechnology are used to increase activities. This figure illustrates various nanotechnology-driven strategies, such as nanohybrids, chitosan nanoparticles, nanoemulsions, and fucoidan complexes, that enhance their anticancer efficacy by promoting apoptosis, increasing ROS production, inducing cell cycle arrest, and improving cellular targeting. These advanced delivery systems significantly contribute to the inhibition of cancer pathogenesis across multiple tumor types. The upward arrow represents upregulation.

Rutin’s solubility, bioavailability, and activity are increased by various nanotechnology-based methods (Figure 4 and Table 3). Tablets containing rutin nanocrystals that had been lyophilized were assembled, and the tablets’ dissolving characteristics were investigated. The tablet loaded with rutin nanocrystals had a faster disintegration rate than the tablet that was loaded with rutin nanocrystals. Rutin was liberated and dissolved in water from the nanocrystal tablets after 30 min. However, only 71 and 55%, respectively, of the total amount of rutin was dissolved from the marketed tablet and the microcrystal tablets. The body’s bioavailability of the insoluble rutin is anticipated to increase due to the tablet’s increased solubility, which contains rutin nanocrystals [206].

**Table 3:** Nanoformulation and its effects on cancer through *in vivo* and *in vitro* analysis

Nanoformulation	Cancer types	Study types	Findings of the study	Refs.
MgO-NH-PBA-Rutin nanohybrids	Breast	*In vitro* (MDA-MB-231)	Induced apoptosis and migration in cancer cells via generating intracellular ROS and demonstrated the most anticancer effects	[113]
Chitosan nanoparticles (CNPs) loaded with rutin	Liver	*In vitro* (Hep3B cells)	Showed significant lethality to cancer cells and excellent hemocompatibility	[209]
Rutin-loaded PLGA NPs	Liver	*In vivo* (albino male rats)	Prevented the formation of nodules in the hepatic tissue, reduced total bilirubin, increased albumin and total protein, and displayed a lower frequency of hepatic nodules, decreased NF-κB level	[210]
Rutin-chitosan nanoconjugates	Breast	*In vitro* (MDA-MB-231)	Enhanced activity against cancer cells, cell cycle arrest at DNA synthesis phase	[211]
Chitosan/poly (acrylic acid) nanogel)	Liver	*In vivo* (Male albino rat)	Improved anti-proliferative, apoptotic, and anti-angiogenic effects	[212]
Chitosan/copper oxide (CS–CuO) nanocomposite using rutin	Lung	*In vitro* (A549 cells)	Nanocomposite exhibited concentration-dependent anti-proliferative potential, induced apoptosis	[213]
Encapsulated FA conjugated keratin NPs	Breast	*In vitro* (MCF-7 cells)	Increased ROS levels, cell death	[214]
Rutin–fucoidan (Ru–Fu) complex	Prostate	*In vitro* (PC3cells)	Disrupted cell cycle regulation, induced cellular apoptosis	[133]
Rutin nanoemulsion	Prostate	*In vitro* (PC3 cells)	Showed significant efficiency against cancer cells	[215]

An earlier investigation was conducted to design and assess a solid self-emulsifying drug delivery system (SSEDDS) as a potential rutin carrier. Liquid SEDDS was created following the screening of several carriers (oils, surfactants, and co-surfactants) and selecting those with superior drug solubilizing power. Neusilin^®^ US2: S6 (1:2) in the SSEDDS (SS4) produced the best drug release characteristics. In summary, the optimal liquisolid dosage form of rutin demonstrated good flowability and rapid drug release properties, making it a potential candidate for oral administration systems [207]. In order to create rutin-containing nanomedicines, Almeida and colleagues produced rutin-loaded nanocapsules and nanoemulsions as aqueous intermediates or final systems. Improved rutin photostability and extended *in vitro* antioxidant activity were demonstrated by both formulations [208].

Different types of nanoformulation are used to evaluate their effects on cancer (Table 3). To create MgO-NH-PBA-Rutinnanohybrids, new PBA-tagged MgO nanoparticles were engineered to target human breast cancer cells. Rutin was then loaded into the nanoparticles. In addition to inhibiting MDA-MB-231 cell migration and death, the MgO-NH-PBA-Rutin nanohybrid exhibited significant anticancer potential against MDA-MB-231 cells by inducing intracellular ROS production. Furthermore, *in vivo* investigations demonstrated the nanohybrid’s more substantial anticancer effects in mice with tumors. However, when compared to both MgO-NH-PBA and free rutin, this formulation had the most anticancer effects in both circumstances [113].

Rutin-loaded chitosan nanoparticles (rCNPs) were synthesized and confirmed to have a cytotoxic effect on human hepatoma Hep3B cells. It was observed that the particles exhibited significant hemocompatibility and were highly cytotoxic to Hep3B cells compared to normal cells. Additionally, variations in the potential of the mitochondrial membrane (MMP) and elevated generation of ROS were observed [209]. After creation, the Rutin-loaded PLGA NPs (RT-PLGA-NPs) underwent an activity assessment. With RT-PLGA-NP treatment, it was observed that this formulation demonstrated a significant downregulation of proinflammatory cytokines and a decreased incidence of hepatic nodules, inflammatory cell infiltration, necrosis development, cell swelling, and blood vessel inflammation [210].

The rutin–chitosan nano-conjugates were made, and these exhibit remarkable anti-breast cancer cell activity. Furthermore, it was observed that after apoptotic cell death, cell cycle arrest occurs at the DNA synthesis phase. The results of this study confirm that designed nano-conjugates could effectively trigger the apoptotic process in triple-negative breast cancer cells [211]. Activity was assessed after producing chitosan/poly (acrylic acid) nanogel. The developed formulation exhibited significantly improved anti-proliferative, apoptotic, and anti-angiogenic effects compared to free rutin. This suggests that the formulation may offer a novel therapeutic approach as a promising agent for treating hepatocellular cancer [212].

Another study used rutin to generate an eco-friendly, bioinspired chitosan/copper oxide (CS–CuO) nanocomposite. The produced CS–CuO nanocomposite exhibited concentration-dependent anti-proliferative activity against A549 cancer cells, as indicated by the results. Furthermore, formulation causes treated cancer cells to undergo apoptosis [213]. After rutin-encapsulated FA-conjugated keratin NPs (FA@Ker NPs) were created, 50% of MCF-7 cancer cells were killed in the formulation. Additionally, the rutin absorption in MCF-7 cells was enhanced by this formulation, and it was noted that the apoptotic potential of deformed membrane bodies and condensed nuclei was present. Additionally, formulation infiltrated the MCF-7 cells’ mitochondria and may have raised the concentration of ROS, which resulted in cell death [214].

The compound known as rutin-fucoidan, or Ru–Fu, is prepared and assessed. The outcome demonstrated that the complex’s formulation may disrupt the control of the cell cycle and cause cellular death by causing nuclear fragmentation, loss of mitochondrial potential, and the production of ROS. Additionally, the complex demonstrates biocompatibility with normal cells [113,122]. In PC3 cancer cells, the IC_50_ value of the improved nanoemulsion formulation was 11.8 μM. Significant ROS induction was shown by fluorescent microscopic examination and intracellular ROS formation, which may start the apoptotic process [215].

## 6 Synergistic effect of rutin with other compounds in cancer management

**Figure 5:**
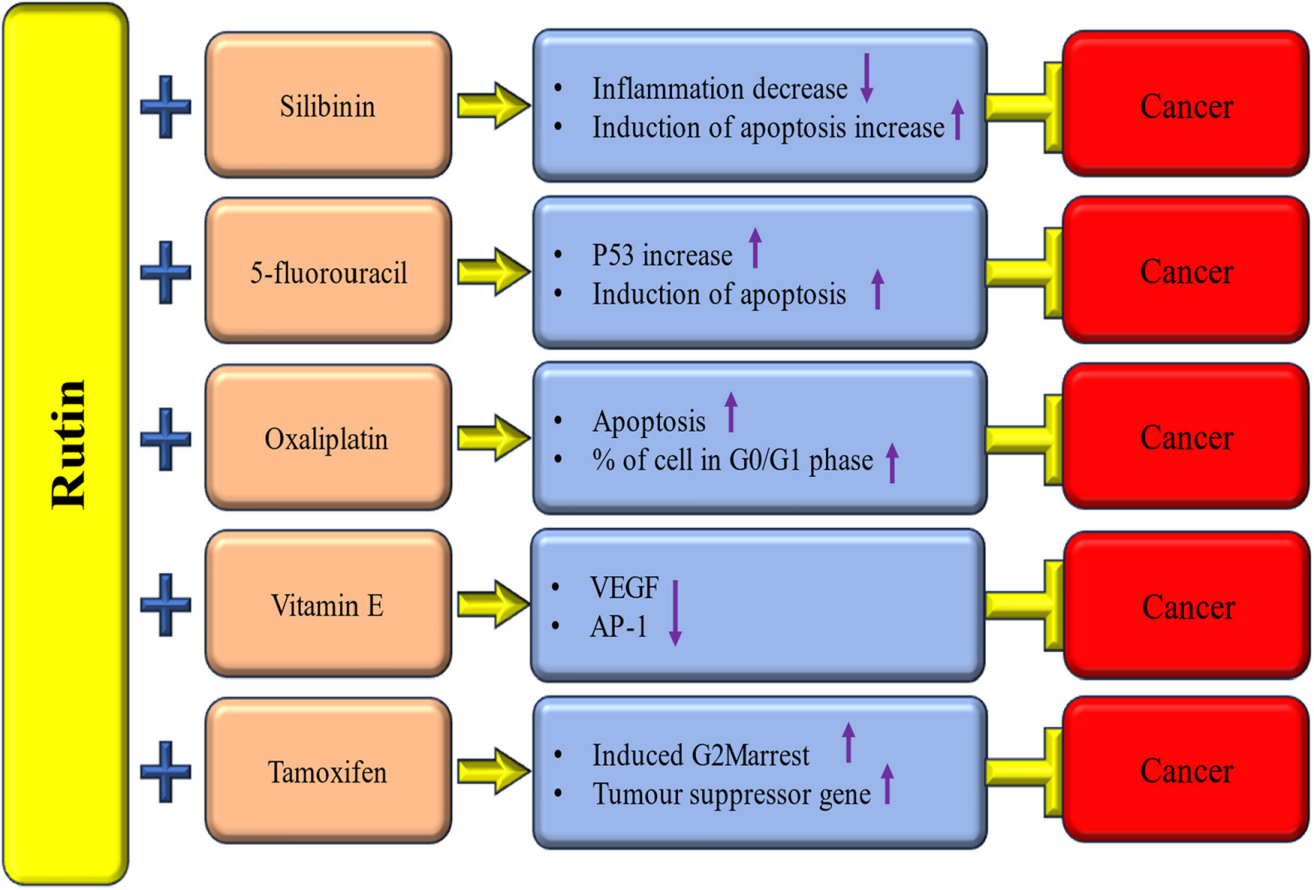
Synergistic effects of rutin with compounds and cancer drugs. Synergistic effects of rutin with various anticancer agents enhance therapeutic outcomes by induction of apoptosis, activation of tumor suppressor genes, cell cycle arrest, inhibition of inflammation, and angiogenesis. These combinations demonstrate rutin’s potential as an adjunct compound in cancer treatment.

Rutin has been widely investigated for its potential to enhance the efficacy of conventional anticancer agents through synergistic interactions with chemicals and anticancer medications (Figure 5 and Table 4). Combination therapy involving rutin aims to reduce drug resistance, minimizes toxicity, and boosts therapeutic efficacy. For example, in the colon cancer model, the combination of silibinin and rutin was shown to induce apoptosis more effectively by modulating the expression of genes associated with inflammation, apoptosis, and MAPK pathway proteins [142]. In prostate cancer cells (PC3 cells), rutin with 5-fluorouracil upregulated p53 gene expression and promoted apoptosis. Further, this combination decreased the formation of PC3 cell colonies and suppressed Bcl-2 signaling protein [42].

**Table 4:** Synergistic effect of rutin with other compounds in cancer

Cancer types	Other compounds	Study types	Cell lines	Outcome	Refs.
Colon cancer	Silibinin	*In vitro*	HT-29	The apoptosis induction and regulation of the expressions of genes associated with inflammation are more efficient in combination than individually	[142]
Prostate cancer	5-fluorouracil	*In vitro*	PC3	Combination of 5-fluorouracil/rutin increased p53 gene expression and apoptosis	[42]
Breast cancer	Tamoxifen	*In vitro*	MCF-7	The rutin synergistically enhanced tamoxifen’s anti-proliferative potential	[43]
Leukemia	Vitamin E	*In vitro*	HL-60	The rutin plus vitamin E synergistically decreases IRS-1 protein expression	[80]
				When vitamin E is combined with rutin, VEGF expression is decreased	
Gastric cancer	Oxaliplatin	*In vitro*	SGC-7901	Apoptosis was significant after combination treatment	[163]
				The greatest notable alteration was produced by the combination therapy	

Similarly, rutin combined with tamoxifen exhibited antiproliferative effects in breast cancer MCF-7 cells. Meanwhile, rutin protects non-cancerous breast MCF-10A cells against tamoxifen toxicity [43]. In human promyelocytic leukemia (HL-60) cells, rutin and vitamin E jointly reduced the expression of the IRS-1 protein, implicating redox-independent mechanisms in their anticancer activity [80].

Another study in gastric cancer SGC-7901 cells demonstrated that cotreatment with rutin and oxaliplatin promoted apoptosis in the cells. Additionally, the cell cycle progression of gastric cancer SGC-7901 cells was evaluated after this combination treatment. The cell cycle arrest at the G0/G1 phase was increased compared to the control cells to variable degrees [116,163].

Despite these promising findings, it has been documented that not all interactions between rutin and chemotherapeutic drugs are beneficial. For example, rutin exhibited antagonism with cytarabine in human leukemia cells, in contrast to other flavonoids like isorhamnetin and kaempferol, which showed synergy. This antagonism was associated with a lack of G2/M phase arrest and may result from rutin’s redox-modulating effects, highlighting the importance of carefully selecting flavonoid combinations based on their molecular interactions [173].

Therefore, while rutin shows considerable promise in enhancing the therapeutic index of anticancer drugs, its use in combination regimens should be guided by careful pharmacodynamic and mechanistic studies. Evaluating both synergistic and antagonistic interactions is essential to ensure efficacy and avoid unintended compromise in treatment outcomes.

## 7 Pharmacokinetics, safety profile, and limitations of rutin in cancer

Rutin is absorbed in the intestine and distributed as quercetin. 35± Because dietary rutin is metabolized by the microbes in the gastrointestinal tract and transformed into other chemicals that can be absorbed, it is rarely or never absorbed in its original form. Rutin is broken down enzymatically by the microbes in the lower gastrointestinal system into its aglycone form, quercetin, and the sugar residue. The epithelial cells that line the small intestine subsequently absorb these resultant metabolites. Rats and rabbits given rutin orally have metabolic changes that produce three rutin metabolites: 3,4-dihydroxyphenylacetic acid, 3-methoxy-4-hydroxyphenylacetic acid, and 3-hydroxyphenylacetic acid. Urine excretion is the next step in the body’s removal of these metabolites [216]. Rats given 328 μmol/kg orally showed quercetin sulfates and glucuronides as the primary metabolites, although rutin was eliminated from the bloodstream [204]. Additional rutin metabolites were also documented, including quercetin, m-hydroxyphenyl-acetic acid, 3,4-dihydroxyphenyl-acetic acid, homovanillic acid, and 3,4-dihydroxytoluene. Roughly 10% of rutin is excreted in urine, with the remaining 10% ending up in feces [217].

In order to verify a sample’s safety during the drug discovery process, toxicity assessment is a crucial prerequisite. Rutin can be deemed safe for additional clinical usage based on the results of previous investigations [218]. Based on earlier research, rutin can be considered safe for ongoing therapeutic use. Rats, guinea pigs, and rabbits were used to test the acute and long-term toxicity of rutin; none of the test animals showed any negative symptoms. After 400 days of this kind of diet, histological examination of the tissues showed no evidence of damage that could be directly attributed to the administration of rutin, and even 1% of rutin in the diet did not slow down the growth rate of albino rats. The organ weights of the test animals fell within typical bounds. S. Rats given a 1% rutin diet reproduced well, produced offspring that looked healthy, and had an estrous cycle that was the same length as control animals. By the standards employed, rutin poses no immediate or long-term risks. Furthermore, up to 50% of the 75 mg of rutin that had been consumed was due to microbial metabolites discovered in human urine [217].

Intestinal enzymes are unable to effectively break down rutin after oral ingestion. However, the gut microbiota can break down rutin through the enzymes β-rutinosidase and α-rhamnosidase. These enzymes convert therutin to aglycones, or genins, which the intestinal epithelial cells can absorb to a certain degree. The gut microbiota greatly enhances rutin metabolism, but intestinal epithelial layers still only absorb a small amount of it. Because of its hydrophobic molecular structure, rutin’s use as a bioactive molecule in biomedical applications is limited. Reduced absorption and, as a result, lower bioavailability of this substance are caused by insufficient solubility in aqueous systems. Other drawbacks of rutin include its short half-life, limited thermal stability, and quick metabolism, all of which can lessen its pharmacological benefits [219].

## 8 Conclusion and future direction

The primary treatment modalities for cancer, including surgery and chemotherapy, often lead to unwanted side effects and drug resistance. Therefore, it is essential to explore safe and effective cancer treatment drugs. Previous investigations support the effectiveness of rutin in preventing and managing various types of cancers. However, rutin faces challenges in clinical development due to its low solubility and limited oral bioavailability. Nano-formulations of rutin have been shown to improve bioavailability and increase efficacy in various cancer cell lines.

Currently, there is no large-scale clinical trial dealing with rutin as a standalone anticancer agent in humans. Most of the evidence for rutin’s anticancer potential comes from *in vitro* studies and animal models, where it has been shown to induce apoptosis, anti-angiogenesis potential, and anti-metastasis activity across various cancer types. Pharmacological limitations and a lack of clinical trials hinder its clinical translation. Advances in nanotechnology-based delivery systems and better clinical trial design could help bridge this gap in the future.

Future research should focus on comprehensive human studies, mechanistic exploration in diverse tumor microenvironments, and the development of advanced targeted nanoformulations. Furthermore, additional research is needed to investigate the mechanism of action and safety of rutin. Additionally, combination therapy involving rutin and existing chemotherapeutic drugs may offer synergistic effects while reducing side effects and toxicity.
